# CHI3L1 on fibrinolytic system imbalance in chronic rhinosinusitis with nasal polyp

**DOI:** 10.3389/fimmu.2024.1410948

**Published:** 2024-06-21

**Authors:** Hyun-Woo Yang, Joo-Hoo Park, Jae-Min Shin, Hyeong-Guk Son, Tae-Hoon Kim, Seung-Hoon Lee, Il-Ho Park

**Affiliations:** ^1^ Upper Airway Chronic Inflammatory Diseases Laboratory, Korea University College of Medicine, Seoul, Republic of Korea; ^2^ Department of Otorhinolaryngology-Head and Neck Surgery, Korea University College of Medicine, Anam Hospital, Seoul, Republic of Korea; ^3^ Medical Device Usability Test Center, Guro Hospital, Korea University College of Medicine, Seoul, Republic of Korea; ^4^ Department of Otorhinolaryngology-Head and Neck Surgery, Korea University College of Medicine, Ansan Hospital, Ansan, Republic of Korea; ^5^ Department of Otorhinolaryngology-Head and Neck Surgery, Korea University College of Medicine, Guro Hospital, Seoul, Republic of Korea

**Keywords:** Chitinase-3-like protein 1, chronic rhinosinusitis, fibrinolytic system, plasminogen activator inhibitor-1, tissue plasminogen activator

## Abstract

**Background:**

Chronic rhinosinusitis (CRS) is an inflammatory disease affecting more than 10% of the global adult population. It is classified into Th1, Th2, and Th17 endotypes and eosinophilic and non-eosinophilic types. Th2-based inflammation and eosinophilic CRS (ECRS) are associated with tissue remodeling and fibrinolytic system impairment.

**Objective:**

To elucidate the role of eosinophils in inducing fibrin deposition in CRS nasal polyp tissues and explore potential regulatory mechanisms.

**Methods:**

We analyzed the expression of genes related to the serpin family and fibrinolytic system using Gene Expression Omnibus and Next-generation sequencing data. Differentially expression genes (DEGs) analysis was used to compare control and nasal polyp tissues, followed by KEGG and Gene ontology (GO) analysis. We measured the expression and correlation of plasminogen activator-1 (PAI-1), tissue plasminogen activator (t-PA), urokinase plasminogen activator (u-PA), and urokinase plasminogen activator surface receptor (u-PAR) in CRS tissues, and evaluated the effect of eosinophils on the fibrinolytic system using a cytokine array and co-culture.

**Results:**

Nasal polyp tissues showed upregulated PAI-1, u-PA, and u-PAR expression and downregulated t-PA expression. Fibrinolytic system-related genes positively correlated with Th2 cytokines, except for t-PA. Eosinophil-derived Chitinase-3-like protein 1 (CHI3L1) increased PAI-1 expression and decreased t-PA levels in fibroblasts and epithelial cells. The inhibition of CHI3L1 suppresses these alterations.

**Conclusion:**

CHI3L1 contributes to fibrin deposition by impairing the fibrinolytic system during nasal polyp formation. The regulation of CHI3L1 expression may inhibit fibrin deposition and edema in ECRS, presenting a potential treatment for this condition.

## Introduction

Chronic rhinosinusitis (CRS) is a multifactorial inflammatory disease that affects the nose and the paranasal sinuses. CRS is categorized into eosinophilic CRS (ECRS) and non-eosinophilic CRS (NECRS), based on the ratio of eosinophils in the nasal tissue. ECRS, characterized by an increased tissue eosinophil count, is accompanied by a Th2 inflammatory response and is often associated with allergic diseases. Specific treatments such as corticosteroids are required. In contrast, NECRS, which does not exhibit excessive eosinophilic infiltration, is correlated with Th1 and Th17 responses and may be linked to potential bacterial infections or non-allergic causes. Management of ECRS is particularly challenging, as excessive eosinophilic infiltration is closely linked to the persistence of the disease and the tendency for nasal polyps to recur after endoscopic sinus surgery. The hallmark of ECRS at the tissue level is pronounced edema, making it a more severe and recurrent variant than NECRS (1–3).

The prevalence of ECRS shows geographical and ethnic differences. Eosinophilic infiltration is predominantly observed in Western Caucasian patients with CRS, accounting for more than 80% of all CRS cases (4). In contrast, the eosinophilic endotype accounts for less than 50% of CRS cases in East Asian countries, including Japan, China, and South Korea. Recent studies indicate an increased prevalence of ECRS in Asian countries (5, 6). This upward trend is speculated to be due to the Westernization of lifestyles in Asian countries. For instance, a study comparing the histopathological findings of nasal polyps in Korea between 1993 and 2011 reported a significant increase in the prevalence of eosinophilic nasal polyps from 24.0% to 50.9% (7). The dominance of ECRS and the resulting increase in CRS severity is now a global concern, not just a Western issue.

Eosinophils, granulated innate immune cells, play a pivotal role in type 2 inflammation. Through the mediation of IL-5, they mature and migrate to tissues, fueling inflammatory responses. Upon activation, eosinophils release mediators such as eosinophil cationic proteins, major basic proteins, eosinophil-derived neurotoxins, and eosinophil peroxidases (8). These compounds not only induce inflammation but also damage local tissues, facilitating the formation of nasal polyps. Furthermore, eosinophilic mediators enhance vascular permeability, leading to fluid accumulation and swelling in the nasal mucosa. An eosinophil-rich environment fosters a Th2-dominant response that further influences the progression of nasal polyposis (9).

The hemostatic system acts as the body’s natural defense mechanism, clotting blood when vessels are damaged and preventing excessive bleeding. However, excessive blood coagulation can lead to critical complications, such as clot formation and potential occlusion of vessels. To counteract this, the body has a fibrinolytic system responsible for dissolving clots and restoring blood fluidity. This system breaks down fibrin polymerization and causes excessive fibrin accumulation (10, 11). However, the dysregulation of the coagulation cascade in some pathological conditions can cause several diseases like rheumatoid arthritis, adult respiratory distress syndrome, and severe asthma (12). Tissue-type plasminogen activator (tPA) is an enzyme that converts plasminogen into plasmin and is primarily produced in epithelial cells, endothelial cells, macrophages, and fibroblasts (13). t-PA binds to the fibroblast low-density lipoprotein receptor-related protein-1 receptor (LRP-1R) to enhance the production of collagen and extracellular matrix (ECM) in fibroblasts. In nasal polyps, low t-PA expression can cause fibroblasts to produce insufficient ECM, leading to reduced tissue density (14). Conversely, fibrin deposition occurs in the presence of inadequate plasminogen activation. Such fibrin depositions are predominantly observed in patients with CRS with nasal polyps (CRSwNP), characterized by Th2 dominant inflammation. A previous study reported that the concentration of t-PA in nasal polyp tissues of CRSwNP patients was significantly lower than that in CRSsNP patients or control uncinate process (UP) tissues, indicating an impairment in the fibrinolysis pathway of nasal polyp tissues, leading to excessive fibrin accumulation. Inhibitors of tPA, urokinase-type plasminogen activator (u-PA), and Plasminogen Activator Inhibitor-1 (PAI-1) suppress the fibrinolytic system, leading to fibrin deposition in local tissues (15, 16). Recent research has revealed that PAI-1 is highly expressed in nasal polyps and is closely associated with fibrin deposition, primarily expressed in structural cells, including fibroblasts and epithelial cells (17, 18).

Chitinase 3-like 1 (CHI3L1), also known as YKL-40, is a glycoprotein implicated in various inflammatory conditions and tissue remodeling processes. Elevated levels of CHI3L1 have been observed in several chronic inflammatory diseases, including asthma, where it is thought to contribute to disease pathogenesis through its effects on immune cell recruitment, tissue remodeling, and fibrosis (19–22). Recent studies suggest that CHI3L1 plays a significant role in regulating the fibrinolytic system. Specifically, CHI3L1 upregulates Th2 inflammatory responses, disrupts the Th1/Th2 balance, and promotes the differentiation of macrophages into the M2 type, thereby enhancing fibrin deposition in ECRS. Elevated CHI3L1 levels are found in eosinophilic respiratory diseases like asthma, serving as a biomarker for disease severity (23–26).

While studies have explored the relationship between ECRS and the fibrinolytic system, none have yet demonstrated that structural cells influenced by eosinophils play a role in regulating the key components of the fibrinolytic system. This research will provide new insights into the mechanisms by which eosinophil-derived CHI3L1-induced structural cell changes affect fibrin deposition and may identify novel therapeutic targets for managing ECRS.

## Materials and methods

### Harnessing GEO datasets for microarray and NGS data analysis

Using the public gene expression datasets GSE23552 (Affymetrix Human Exon 1.0 ST Array), GSE36830 (Affymetrix Human Genome U133 plus 2.0 array), and GSE179265 (Illumina NovaSeq 6000) from the GEO database, we studied the gene expression profiles of patients with CRS (27–29). Clinical characteristics of the patients are summarized in **Supplementary Table 1**. For the microarray dataset, background correction was performed to minimize technical bias, and the data were normalized using quantile normalization. For the NGS dataset, raw read counts were normalized using the Transcripts Per Million (TPM) values, which account for both sequencing depth and gene length differences between samples. Additionally, to visualize the data, expression values were log2-transformed, allowing for a more intuitive comparison of gene expression levels across different samples. These processed datasets were then used to identify differentially expressed genes.

### Gene expression analysis and hierarchical clustering

For the hierarchical clustering, R statistical software (version 4.2.2) and the ‘pheatmap’ package (version 1.0.12) were used. The Euclidean distance metric and complete linkage method were chosen to measure the similarity between gene expression profiles and to merge clusters, respectively. Hierarchical clustering was performed on each dataset separately, using the expression profiles of genes related to the serpin family, fibrinolytic system, and inflammatory cytokines and chemokines. Based on the gene expression patterns, samples were divided into two main groups: Cluster 1, which exhibited characteristics of healthy controls, and Cluster 2, which consisted of nasal polyp samples with significantly increased or decreased expression of the target genes. The heatmap generated visualized the relative gene expression levels of the selected genes across all samples. Following the identification of Cluster 1 and Cluster 2, subsequent analyses were performed to further characterize these groups and explore potential molecular mechanisms underlying the observed gene expression patterns.

### Principal component analysis and multi-dimensional scaling

iDEP, an online platform for the integrated analysis of RNA-Seq data, was employed to process the gene expression datasets and perform PCA. The PCA plot generated by iDEP allowed for the visualization of the relationships between samples and the identification of potential outliers. For MDS analysis, the R statistical software (version 4.2.2) and the ‘stats’ package (version 4.2.2) were used. The ‘cmdscale’ function was applied to calculate the MDS coordinates based on the Euclidean distance matrix derived from the log-transformed gene expression data. In the PCA and MDS plots, samples belonging to Cluster 1 and Cluster 2 were distinctly marked with different colors to clearly differentiate the two groups.

### Differential gene expression analysis

To identify differentially expressed genes (DEGs) between the two clusters (Cluster 1 and Cluster 2) of samples, we employed the edgeR and Limma packages in R. The raw count data were processed and normalized using the respective methods for each analysis tool. For edgeR and Limma, DEGs were identified using the generalized linear model (GLM) and linear modeling approaches, respectively. In iDEP, DEGs were determined using the integrated DEG analysis pipeline provided by the software. We filtered the results to obtain genes that met our criteria of |log2 fold change (FC)| > 1 and adjusted an p-value < 0.05. Subsequently, volcano plots were generated to visualize the distribution of DEGs across the samples.

### Functional enrichment and pathway analysis

Utilizing the results of the DEG analysis, we performed Gene Ontology (GO) and Kyoto Encyclopedia of Genes and Genomes (KEGG) pathway analyses using the clusterProfiler package in R. We focused on the GO Biological Process and KEGG pathway analyses, applying a cutoff of 0.05 for the adjusted p-value. To further investigate the target fibrinolytic system, we conducted an additional analysis on the “Complement and coagulation cascades (hsa04610)” pathway. Within this pathway, we annotated the expression of individual genes.

### Human participants

UP and nasal polyp tissues were obtained from 53 patients (28 males and 25 females). Normal UP tissues were harvested during septoplasty. Nasal polyp tissues were obtained from the middle meatus at the beginning of the endoscopic surgical procedure in patients with CRS. CRS was diagnosed based on historical, endoscopic, radiographic, and computed tomography (CT) findings of the sinuses according to the 2020 European position paper on rhinosinusitis and nasal polyps (EPOS) guidelines (2). All participants were recruited from the Department of Otorhinolaryngology, Korea University Medical Center. Informed consent was obtained in accordance with the principles of the Declaration of Helsinki. This study was approved by the Institutional Review Board of Korea University Medical Center (2020GR0308). Clinical characteristics of the patients are summarized in **Supplementary Table 1**.

### EOL-1 differentiation and cytokine analysis

EOL-1 cells were purchased from Sigma-Aldrich (St. Louis, MO, USA) and cultured in RPMI-1640 medium supplemented with 10% fetal bovine serum (FBS) and 1% penicillin and streptomycin (10,000 U/mL). EOL-1 cells were seeded at a density of 1x10^6^ cells in a 100mm culture dish and 5x10^5^ cells in a 60mm culture dish. To differentiate into eosinophils (differentiated EOL-1; diEOL-1), EOL-1 cells were treated with IL-3 (1 ng/mL), IL-5 (1 ng/mL), GM-CSF (0.5 ng/mL), and butyric acid (BA, 0.5 mM) for 5 days. Flow cytometry was used to ascertain EOL-1 differentiation. After harvesting EOL-1 and diEOL-1 cells, they were fixed with 4% paraformaldehyde for 10 minutes and permeabilized with 0.1% Triton X-100 for 10 minutes at room temperature. After blocking with 1% BSA, the cells were treated with fluorescence-tagged antibodies (CCR3-PE and IL5Ra-FITC). Using microscopy, we confirmed that diEOL-1 was present in the nuclear bilobes, which are characteristic of eosinophils, and FACS data confirmed that the eosinophil markers CCR3 and IL-5Ra were simultaneously expressed. The cytokine expression of EOL-1 and diEOL-1 was assessed using the Proteome Profiler™ Cytokine Array (R&D systems, Minneapolis, USA). Membranes were blocked using blocking buffer and incubated for 1 hour on an orbital shaker at room temperature. After washing, supernatants diluted 1:2 obtained from EOL-1 and diEOL-1 were added to each membrane. The membranes were incubated overnight at 4°C on an orbital shaker. The next day, the array membranes were thoroughly washed with wash buffer, followed by the addition of a detection antibody cocktail to each membrane and incubation at room temperature for 1 hour. After washing, Streptavidin-HRP was applied and incubated for 30 minutes at room temperature. Subsequently, chemiluminescent detection reagents were used for the reaction, and the results were captured on x-ray film. The resultant mean spot pixel density profiles were analyzed using Quantity One software.

### Primary nasal fibroblast and fibroblast spheroid culture

Fibroblasts were isolated from inferior turbinate tissues by enzymatic digestion with collagenase (500 U/mL; Sigma-Aldrich, St. Louis, MO, USA), hyaluronidase (30 U/mL, Sigma), and DNase (10 U/mL, Sigma). Cells were cultured in DMEM supplemented with 10% FBS, 1% penicillin, and streptomycin. The purity of primary fibroblasts was verified using microscopy and flow cytometry. The cultured fibroblasts exhibited spindle-shaped cell morphology, and more than 95% of the fibroblasts expressed vimentin and Thy-1 (Santa Cruz Biotechnology, Santa Cruz, California), which are used as fibroblast markers. For the generation of fibroblast spheroids, we utilized a U-bottom plate (CLS4515, Corning, NY, USA). This U-bottom plate surface is coated with a covalently bonded hydrogel to minimize cell attachment. The fibroblasts were seeded into a 96-well U-bottom plate at a density of 1x10^4^ cells per well and were subsequently cultured for 14 days. The fibroblast spheroids were cultured in each well with 200 μl of DMEM supplemented with 10% FBS, 1% penicillin, and streptomycin. After seeding the fibroblasts, media changes were performed every three days with care to avoid dislodging the spheroids. The average size of the generated fibroblast spheroids, as observed under a microscope, was 250–300 μm in both length and width.

### Primary nasal epithelial cell (PNECs) culture and air-liquid interface culture

Primary nasal epithelial cells (PNECs) were collected using the intranasal brushing method, a noninvasive technique to obtain intranasal epithelial cells. This method allowed collection of basal cells for culture. Collected PNECs were initially cultured on 35 mm plates coated with collagen type I. The culture medium used was Complete PneumaCult™-Ex Basal Medium Plus supplemented with PneumaCult™-ALI 10X supplement, hydrocortisone, and gentamicin at 50 μg/ml. The cells were then subcultured into T-75 flasks and maintained for 3–5 days.

To establish air-liquid interface (ALI) cultures, PNECs were seeded in the upper chamber of a 12-well Transwell^®^ plate at a density of 1x10^5^ cells per well. Cells were cultured with PneumaCult™-Ex Basal Medium Plus, and the medium was changed every 2 days. After 4–5 days, cells were ready to differentiate at ALI. The medium in the basal chamber was then replaced with PneumaCult™-ALI medium, and the medium in the apical chamber was removed to expose the cells to air to initiate the air-liquid interface. The cells were then cultured for an additional 3 weeks and the medium in the basal chamber was changed every 2 days. After 3 weeks, cells were observed to form cilia and mucus.

### Co-culture and 3D hybrid culture system

To establish a co-culture system, EOL-1 cells and primary nasal fibroblasts were utilized. Both EOL-1 cells differentiated only with BA and EOL-1 cells differentiated and activated with BA, IL-3, IL-5, and GM-CSF were co-cultured with fibroblasts. For the Fibroblasts + diEOL-1 group, diEOL-1 cells were added to the cultured fibroblasts. The Fibroblasts + diEOL-1 + C.M group included both diEOL-1 cells and 1 ml of conditioned media generated during the diEOL-1 process. For the ALI culture and diEOL-1 co-culture, diEOL-1 cells were cultured in the lower chamber of the transwell. The conditioned media + condition involved adding 1 ml of conditioned media to the ALI media. Each co-culture was performed with a ratio of 1 for fibroblasts or PNECs to 4 for diEOL-1 cells. The 3D hybrid culture system was performed using ALI culture, EOL-1 differentiation and activation, and Fibroblast spheroid culture included in the method. ALI culture was carried out for 14 days, and it was verified that basal cells differentiated into ciliated epithelial cells. In addition, CCR3+ and IL-Ra+ EOL-1 were sorted from diEOL-1 differentiated for 5 days. Fibroblast spheroids were formed in a 96-well U-bottom plate. The spheroid had a length and width of approximately 250–300 uM. In ALI culture, the upper chamber was inserted into another 24-well plate, and fibroblast spheroids and diEOL-1 were cultured in the lower part. Epithelial cells were 1x10^5^, diEOL-1 was 4x10^5^, and 35–40 spheroids produced in a 96-well plate were used.

### Real-time PCR and ELISA

After co-culture, the supernatant was collected and centrifuged to isolate diEOL-1, while fibroblasts were washed and RNA was extracted using Trizol reagent. cDNA synthesis was performed using M-MLV for both fibroblasts and diEOL-1. Amplification was carried out with denaturation at 94°C for 5 seconds, annealing and elongation at 60°C for 15 seconds, for a total of 40 cycles using QuantStudio 3 (Applied Biosystems, Foster City, CA, USA). Gene expression was analyzed using the 2-ΔΔCT method with GAPDH as the control. Primer details are provided in **Supplementary Table 2**. Proteins secreted by epithelial cells grown in ALI culture were collected from the media in the lower chamber of the Transwell and analyzed by ELISA. The total protein amount was quantified using the Bradford method. Target protein expression, specifically PAI-1, t-PA, and CHI3L1, was assessed using DuoSet ELISA kits. After overnight incubation with capture antibodies, 200 µL of sample media was added to each well, followed by the detection antibody and streptavidin-HRP. The optical density was measured spectrophotometrically.

## Results

### Comparative analysis of fibrinolytic system and cytokine-related gene expression patterns in chronic rhinosinusitis patients

**Figure 1:**
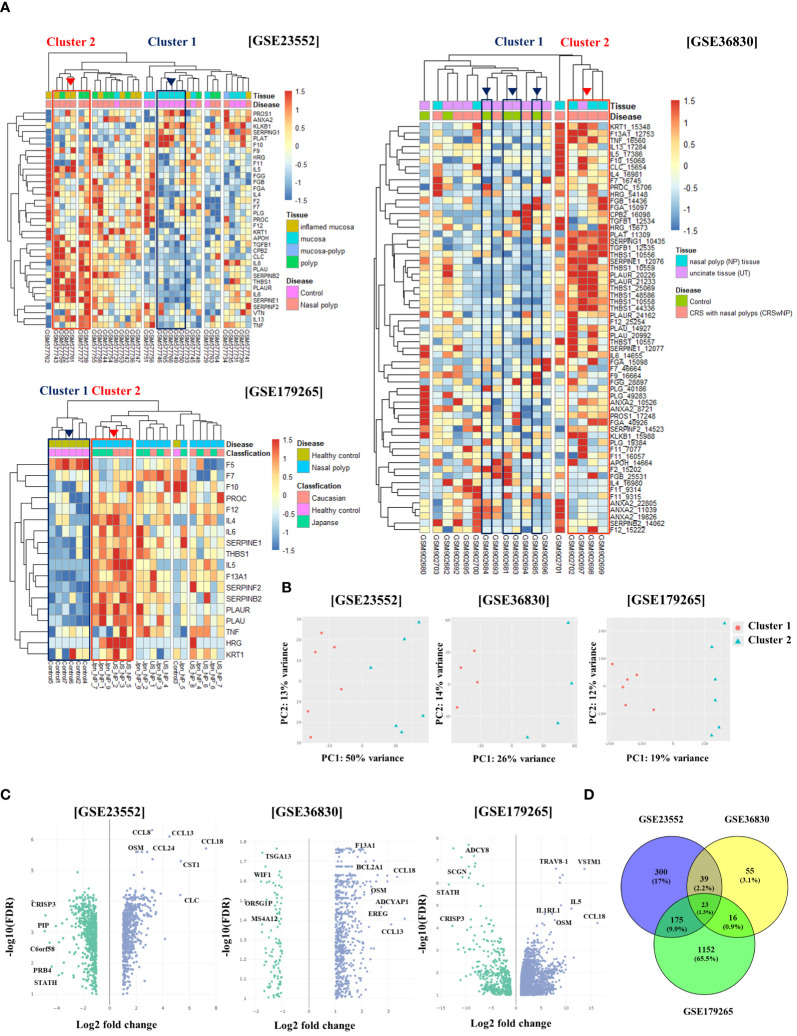
Differential gene expression and clustering patterns in normal and chronic rhinosinusitis nasal tissues. **(A)** Heatmaps showing the expression levels of genes associated with the fibrinolytic system and cytokines across three GEO datasets (GSE23552, GSE36830, and GSE179265). Normal tissue samples are grouped within Cluster 1 (indicated by a blue box), whereas samples from chronic rhinosinusitis with fibrinolytic system imbalance are grouped within Cluster 2 (indicated by a red box). **(B)** Principal Component Analysis (PCA) results depicting Cluster 1 as red circles and Cluster 2 as blue triangles. **(C)** Volcano plots of gene expression derived from differential expression analysis between Cluster 1 and Cluster 2, with the x-axis showing the log2 fold-change and the y-axis displaying the negative log10 of the false discovery rate (FDR). **(D)** A Venn diagram illustrating the overlap of genes identified in the differential expression analysis across the three GEO datasets.

In this study, we examined the expression patterns of fibrinolytic system-related genes in patients with CRS using the GSE23552, GSE36830, and GSE179265 datasets. Data analysis was conducted following the procedure described in **Supplementary Figure 1**. Genomic data were classified into several clusters based on the expression distribution of serpin family-, fibrinolytic system-, cytokine-, and chemokine-related genes. We identified one cluster with an overexpression of fibrinolytic system-related genes (cluster 2), such as SERPINE1 (PAI-1), PLAU (u-PA), and PLAUR (u-PAR), compared to the normal cluster (cluster 1). In contrast, the expression of PLAT (t-PA) was reduced compared with that in the normal cluster. This cluster also displayed the overexpression of Th2 cytokines and CLC. Notably, five samples from the GSE23552 dataset belonging to this cluster were obtained from patients with asthma and aspirin sensitivity (**Figure 1A**). The PCA results for each dataset demonstrated significant clustering by sample, corroborating distinct gene expression patterns (**Figure 1B**). Based on clustering analysis, we performed DEG analysis on the normal cluster (cluster 1) and targeted cluster (cluster 2). In the GSE23552 dataset, the expression of CCL8, CCL13, CCL18, CCL24, OSM, CST1, and CLC was significantly increased. In contrast, the expression of CRISP3 and PIP was decreased. In the GSE36830 dataset, the expression of CCL13, CCL18, OSM, and F13A1 was increased, whereas that of WIF1, OR5GIP, MS4A12, and WIF1 was decreased. In the GSE179262 dataset, the expression of VSTM1, CCL18, OSM, IL1RL1, and IL-5 was increased, whereas that of SCGN, STATH, CRISP3, and LPO was decreased (**Figure 1C**). DEG analysis of the three datasets revealed 23 upregulated genes (**Figure 1D**).

Regarding the CRS endotypes in the GEO Database utilized in **Figure 1**, for GSE23552, the Nasal polyp group are ECRS with aspirin-exacerbated respiratory disease (AERD). In the pheatmap of **Figure 1**, Cluster 2 represents a group of ECRS patients with elevated expression of genes related to the fibrinolytic system (27). In the GSE36830 dataset, it is stated that polyp tissues from CRSwNP patients have significantly increased expression of ECP, IL-5, IL-13, and Eotaxin-1, 2, and 3 compared to normal tissues, indicating eosinophilia. This suggests that the tissues used in our analysis possess characteristics of ECRS (28). The GSE179265 dataset comprises data from both Type 2 and non-Type 2 CRS patients. The original study demonstrates that CST-1, CCL13, CCL18, and CCL26 are upregulated in Type 2 CRS, along with increased expression of IL-5 and IL-13. In the heatmap data from GSE179265 in **Figure 1**, Cluster 2 shows an upregulation of IL-5, a hallmark cytokine of Type 2 inflammation. Moreover, genes that are highly expressed in Type 2 CRS in the original study are also upregulated in Cluster 2. While individual clinical information for each tissue sample is not available due to the nature of external clinical data, the gene expression patterns allow us to infer that the tissues in Cluster 2 belong to patients with Type 2 CRS. Therefore, we can deduce that Cluster 2 in **Figure 1** represents tissues with ECRS or Type 2 inflammatory response (29).

### GO and KEGG analysis reveal immune response activation and fibrinolytic system alterations in chronic rhinosinusitis

**Figure 2:**
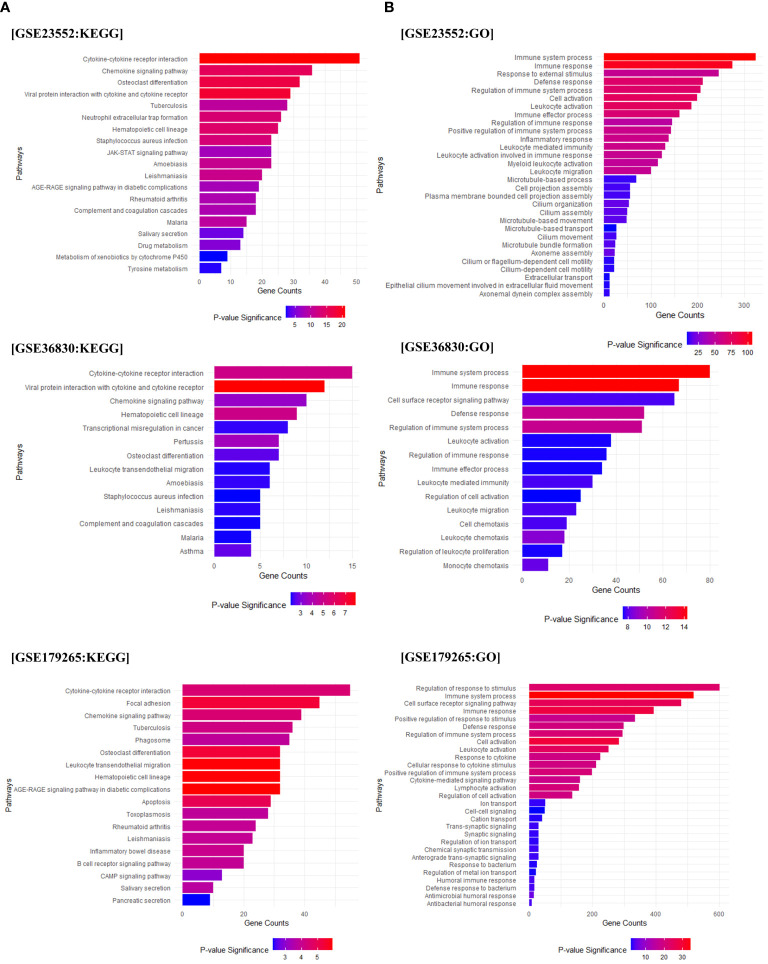
Pathway enrichment analysis. Gene Ontology (GO) and KEGG pathway analysis based on the differentially expressed genes (DEGs) between Cluster 1 and Cluster 2. **(A)** The results of the KEGG analysis for Cluster 2 compared to Cluster 1 (based on Cluster 2). **(B)** Displays the GO analysis results for Cluster 2 compared to Cluster 1 (based on Cluster 2). The X-axis represents the count of genes included in each pathway, and the significance of each pathway is indicated using red and blue indicators.

To investigate the pathways associated with the observed genetic differences, we conducted GO and KEGG analysis. We found pathways related to immune responses, such as hematopoietic cell lineage, cytokine, chemokine signaling pathway, Th1 and Th2 cell differentiation, and neutrophil extracellular trap formation in Cluster 2. A strong correlation was observed for *Staphylococcus aureus* infection, which was associated with the onset of chronic rhinosinusitis. The fibrinolytic system-related pathway and complement and coagulation cascades were upregulated (**Figure 2A**, **Supplementary Table 3**). Gene Ontology (GO) analysis of the biological processes revealed a substantial increase in genes related to immune system processes and regulation, immune responses to external antigens, and leukocyte activation and migration in Cluster 2 (**Figure 2B**). As a result of examining detailed gene expression patterns of the complement and coagulation systems for each data set through KEGG analysis, it was confirmed that the expression of PAI-1, which promotes fibrin deposition in the coagulation cascade pathway, was upregulated. On the other hand, the expression of t-PA, which promotes fibrin degradation, was confirmed to be reduced. In addition, the expression of other fibrinolytic system regulatory genes, u-PA and u-PAR, was also confirmed to be upregulated (**Supplementary Figure 2**).

### Expression of SERPINE1 and PLAT in nasal mucosal structural cells

**Figure 3:**
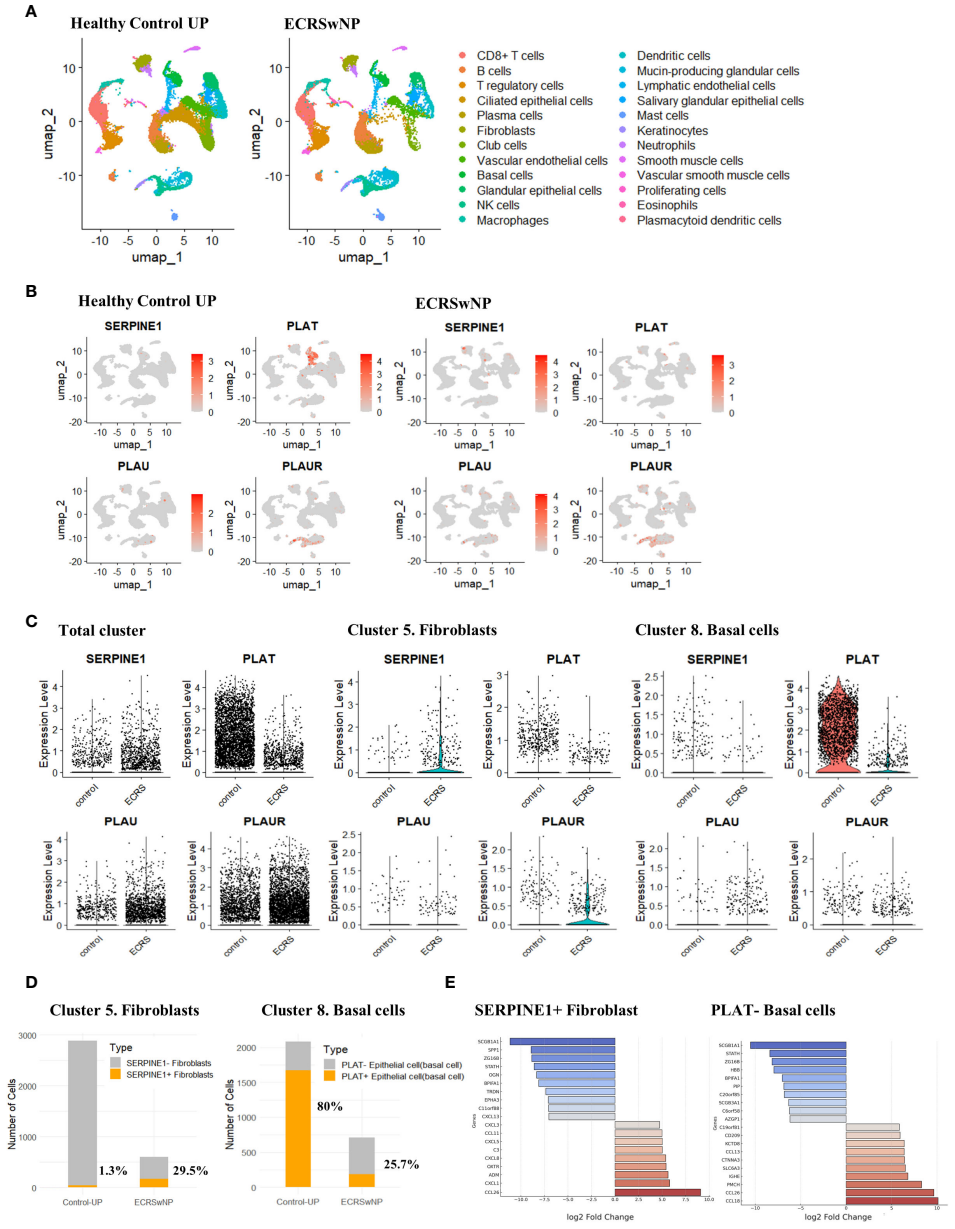
Single-cell transcriptomic profiling and differential expression analysis in ECRSwNP-NP and healthy control tissues. **(A)** Single-cell classification using the Allfindmarker tool. Annotation was performed utilizing the sc-type package and cellmarkers2.0 database. The single-cell patterns in Healthy Control UP and ECRSwNP-NP tissues are presented using UMAP. **(B)** Cellular expression of SERPINE1, PLAT, PLAU, and PLAUR depicted through Featureplot. Expression patterns are differentiated between Healthy Control UP and ECRSwNP NP tissues. **(C)** Vinplot data for the expression of SERPINE1, PLAT, PLAU, and PLAUR. **(D)** Graphs depicting the proportion of cells expressing SERPINE1 in the Fibroblast cluster and PLAT in the Basal cells cluster. **(E)** Boxplots showing the top 10 genes upregulated or downregulated in cells expressing SERPINE1+ fibroblasts and in PLAT- Basal cells.

We performed a single-cell analysis using 5 healthy control UP and 6 ECRS nasal polyp tissue datasets from the single-cell sequencing data of Wang et al. (30) (**Supplementary Figure 3**). We determined the expression of genes associated with the fibrinolytic system in each cell type. SERPINE1 (PAI-1) is mainly expressed in nasal mucosal structural cells such as fibroblasts and epithelial cells. Expression was increased in ECRS compared to normal UP tissue, especially in fibroblasts. In normal UP, the presence of SERPINE1-expressing fibroblasts was only 1.3%. However, in ECRS, 29.5% were confirmed to be SERPINE1 positive despite the fibroblast population being reduced to one-sixth of normal. Conversely, the expression of PLAT (t-PA) in basal cells presented an inverse pattern. In normal UP tissues, 80% of PLAT-positive basal cells was observed. In contrast, ECRS tissues exhibited a marked decrease in PLAT expression, with only 25.7% of basal cells being PLAT-positive (**Figures 3A-D**). Furthermore, SERPINE1-positive fibroblasts in ECRS demonstrated a significant upregulation of genes such as CCL26. Conversely, the expression of genes like SCGB1A1 and SPP1 was found to be reduced in these cells. In PLAT-negative basal cells, genes such as CCL18 and CCL26 were overexpressed, whereas the levels of SCGB1A1 and STATH were diminished (**Figure 3E**, **Supplementary Table 4**).

### Fibrinolytic system-related protein expression in chronic rhinosinusitis tissues and association with eosinophilic airway diseases

**Figure 4:**
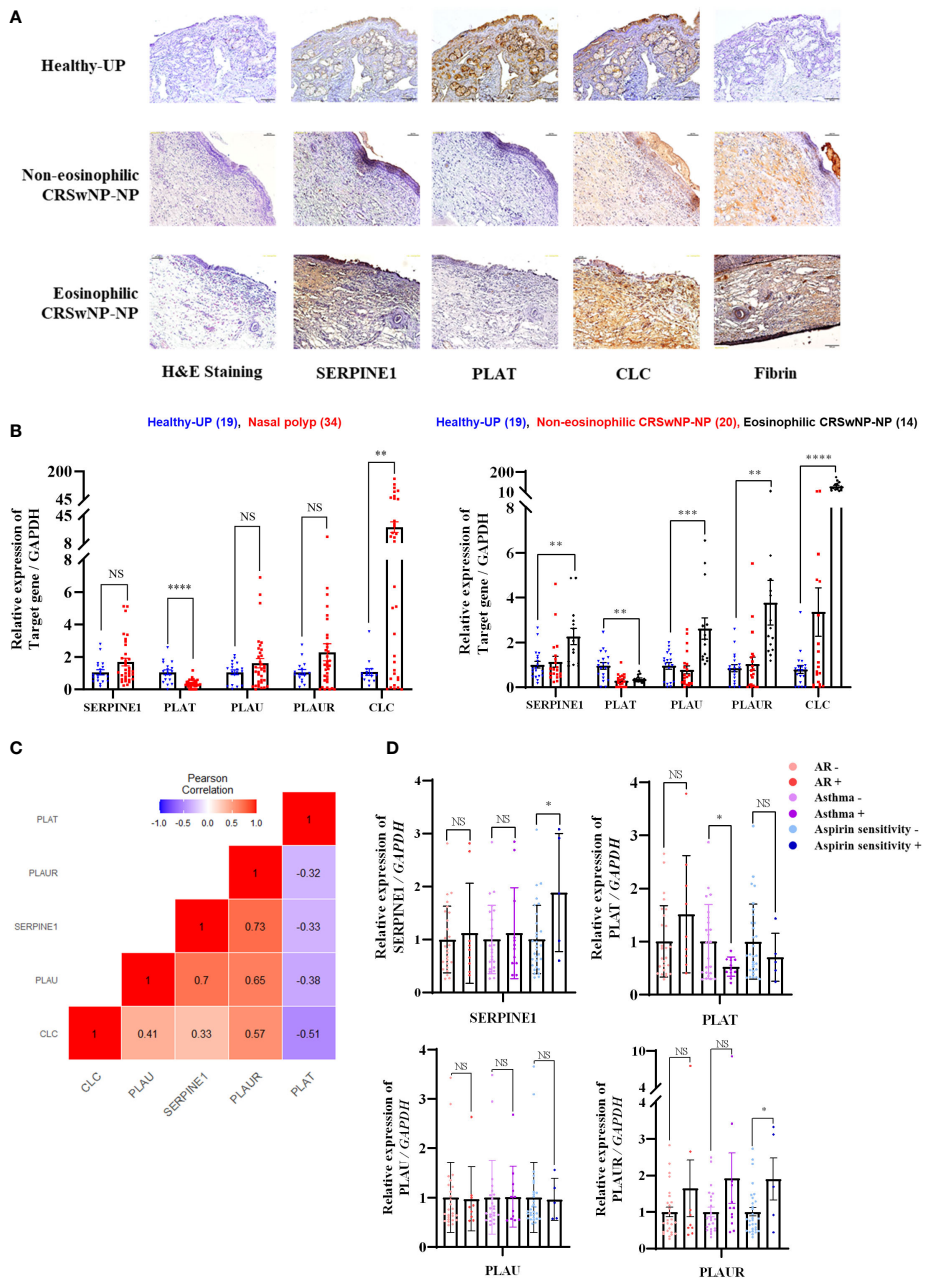
Expression patterns of fibrinolytic system-related genes in sinonasal tissue and their correlation with eosinophilic diseases **(A)** Protein expression of SERPINE1, PLAT, CLC, and Fibrin in sinonasal tissues: Healthy-UP, Non-eosinophilic CRSwNP NP, and Eosinophilic CRSwNP NP, as shown by Immunohistochemistry. **(B)** Gene expression analysis across each tissue group using Real-time PCR. **p < 0.01 vs. Healthy-UP; ***p < 0.001 vs. Healthy-UP; ****p < 0.0001 vs. Healthy-UP. **(C)** Correlation analysis of gene expression. **(D)** SERPINE1, PLAT, PLAU, and PLAUR gene expression results in relation to allergic rhinitis, asthma, and aspirin resistance, as determined by Real-time PCR. *p < 0.05 vs. non-AR or Aspirin sensitivity.

PAI-1 was overexpressed in the stroma of ECRSwNP-NPs. t-PA was highly expressed in the epithelium, stroma, submucosal gland, and vessels of Healthy-UP, but barely expressed in CRSwNP-NP. CLC exhibited a significant increase in the expression of ECRSwNP-NP, accompanied by fibrin deposition (**Figure 4A**). Gene expression analysis of 19 Healthy-UP and 34 nasal polyp tissues revealed that PAI-1, u-PA, and u-PAR showed a slight but non-significant increase in nasal polyps. In contrast, t-PA decreased and CLC increased in the nasal polyps. When classified into NECRS and ECRS, PAI-1 expression was significantly increased in ECRS. t-PA exhibited a significant decrease in nasal polyp tissue compared to Healthy-UP, but no major difference was observed between NECRS and ECRSwNP tissues. PAI-1, u-PA, u-PAR, and CLC showed substantial increases in ECRS (**Figure 4B**). Correlation analysis revealed positive correlations between PAI-1, u-PA, u-PAR, and CLC. t-PA was negatively correlated with other fibrinolytic system-related genes (**Figure 4C**). To investigate the association with eosinophil-related airway diseases such as allergic rhinitis, asthma, and aspirin sensitivity, patients with each condition were classified and compared for the expression of relevant genes. PAI-1 and u-PAR were highly expressed in patients with aspirin sensitivity. PLAU expression did not change regardless of the presence or absence of disease. However, PLAT levels significantly decreased in patients with asthma. Although t-PA seemed to decrease in patients with aspirin sensitivity, and u-PAR increased in patients with asthma, these data were not statistically significant. (**Figure 4D**).

### The diEOL-1 induces an imbalance of fibrinolytic system-related gene expressions in nasal fibroblasts and PNECs

**Figure 5:**
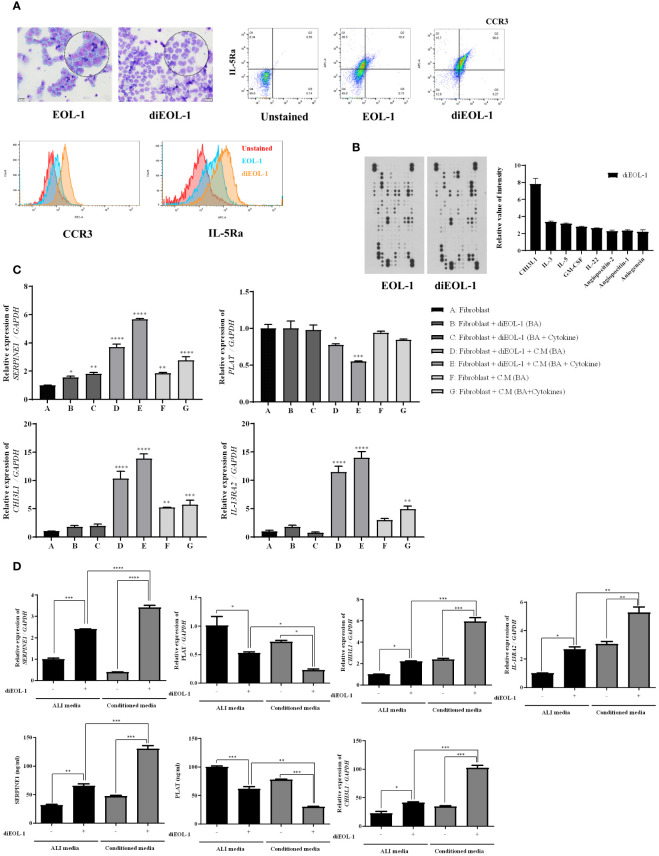
Differentiation of EOL-1 cells and their impact on SERPINE1, PLAT, CHI3L1, IL13RA2 expression in nasal fibroblasts and PNECs EOL-1 cells were differentiated into eosinophil-like cells (diEOL-1) after treatment with IL-3, IL-5, GM-CSF, and butyric acid. **(A)** Representative optical microscope images of H&E-stained EOL-1 and diEOL-1. Dot and histogram graphs of CCR3 and IL-5Ra expression using flow cytometry. **(B)** Human XL cytokine array results and cytokine expression graphs (relative expression increase in diEOL-1 compared to EOL-1). **(C)** Co-culture of fibroblasts and diEOL-1 and the resulting target gene expression analysis using Real-time PCR. *p < 0.05 vs. Fibroblast; **p < 0.01 vs. Fibroblast; ***p < 0.001 vs. Fibroblast; ****p < 0.0001 vs. Fibroblast. **(D)** Target gene expression results from the co-culture of ALI cells and diEOL-1. Expression of SERPINE1, PLAT, CHI3L1 confirmed by ELISA. Asterisks indicate levels of statistical significance: *p < 0.05, **p < 0.01, ***p < 0.001, ****p < 0.0001.

Using microscopy, we confirmed that diEOL-1 was present in the nuclear bilobes, which are characteristic of eosinophils, and FACS data confirmed that the eosinophil markers CCR3 and IL-5Ra were simultaneously expressed (**Figure 5A**). The cytokine array showed the upregulation of CHI3L1 (YKL-40), IL-3, IL-5, and angiopoietin in diEOL-1 cells (**Figure 5B**). After co-culturing nasal fibroblasts with diEOL-1, differentiated and activated diEOL-1 significantly increased the expression of SERPINE1 (PAI-1), CHI3L1, and IL-13RA2 (CHI3L1 receptors) in nasal fibroblasts, whereas it suppressed the expression of PLAT (t-PA). This effect increased when diEOL-1 conditioned media was added during the co-culture process (**Figure 5C**). Similarly, when PNECs cultured in an air-liquid interface (ALI) were co-cultured with diEOL-1, the expression of PAI-1, CHI3L1, and IL-13RA2 was significantly increased compared to that when PNECs were cultured alone, whereas the expression of t-PA was suppressed. This result was even more prominent in diEOL-1 conditioned media. The same trend in protein expression was observed in media obtained after culturing (**Figure 5D**).

### Interaction between diEOL-1 and structural cells of the nasal mucosa causes an imbalance in fibrinolytic system

**Figure 6:**
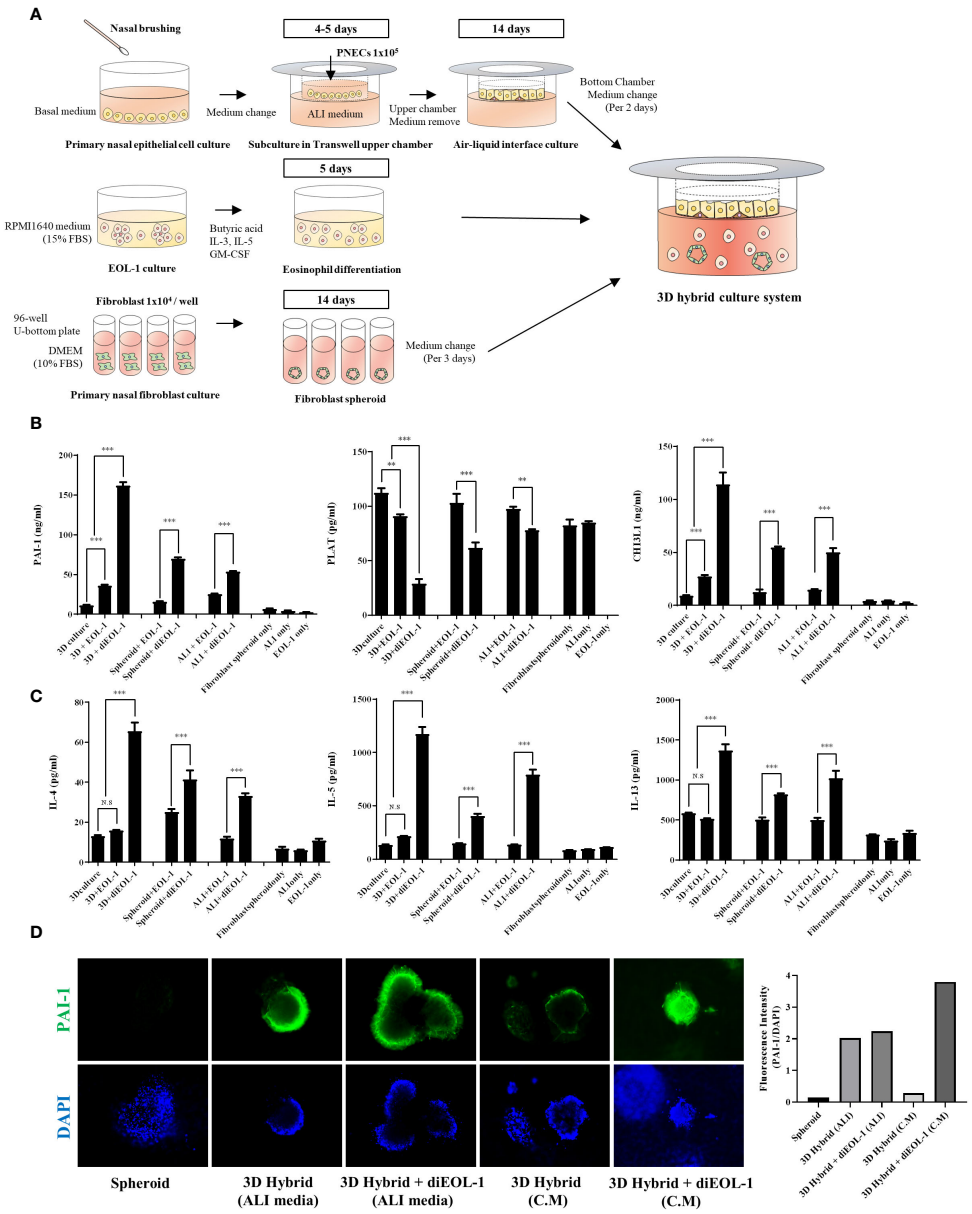
Impact of diEOL-1 on the fibrinolytic system of fibroblasts and ALI cells in a 3D hybrid culture system **(A)** Schematic diagram of the 3D hybrid culture system. **(B)** Protein expression of PAI-1, PLAT and CHI3L1 secreted within the 3D hybrid culture system, as measured by ELISA. Asterisks indicate levels of statistical significance: **p < 0.01, ***p < 0.001, ****p < 0.0001. **(C)** Protein expression of IL-4, IL-5 and IL-13 secreted within the 3D hybrid culture system, determined using ELISA. **(D)** Representative Fluorescence Images: Changes in SERPINE1 (PAI-1) expression in fibroblast spheroids co-cultured with diEOL-1, examined using Immunocytochemistry and confirmed with a confocal microscope (Green: PAI-1, Blue: DAPI).

To explore the effects of diEOL-1 on structural cells in a human-mimicking environment, we executed a 3D hybrid culture system. Using transwell plates, we cultured primary nasal epithelial cells in the upper chamber and fibroblast spheroids in the lower chamber (**Figure 6A**). Subsequently, we co-cultured either EOL-1 or diEOL-1 to validate any alterations in genes related to the fibrinolytic system within the structural cells. When cocultured with EOL-1, enhanced secretion of PAI-1 and CHI3L1 was observed in both ALI and fibroblast spheroids, with diEOL-1 cocultures exhibiting even more substantial increases in secretion. In contrast, tPA secretion was inhibited (**Figure 6B**). The secretion of Th2 cytokines IL-4, IL-5, and IL-13 significantly increased when co-cultured with diEOL-1 cells. As the main cells that originally secrete Th2 cytokines are known to be Th2 cells, additional research is required to determine whether the increased Th2 cytokines through interaction with diEOL-1 and structural cells in this study have relevance and the reason for the increase (**Figure 6C**). The results of fluorescence staining after co-culturing fibroblast spheroids and diEOL-1 revealed overexpression of PAI-1 in spheroids when both cell types coexisted (**Figure 6D**).

### Role of CHI3L1 in the fibrinolytic system imbalance in nasal structural cells

**Figure 7:**
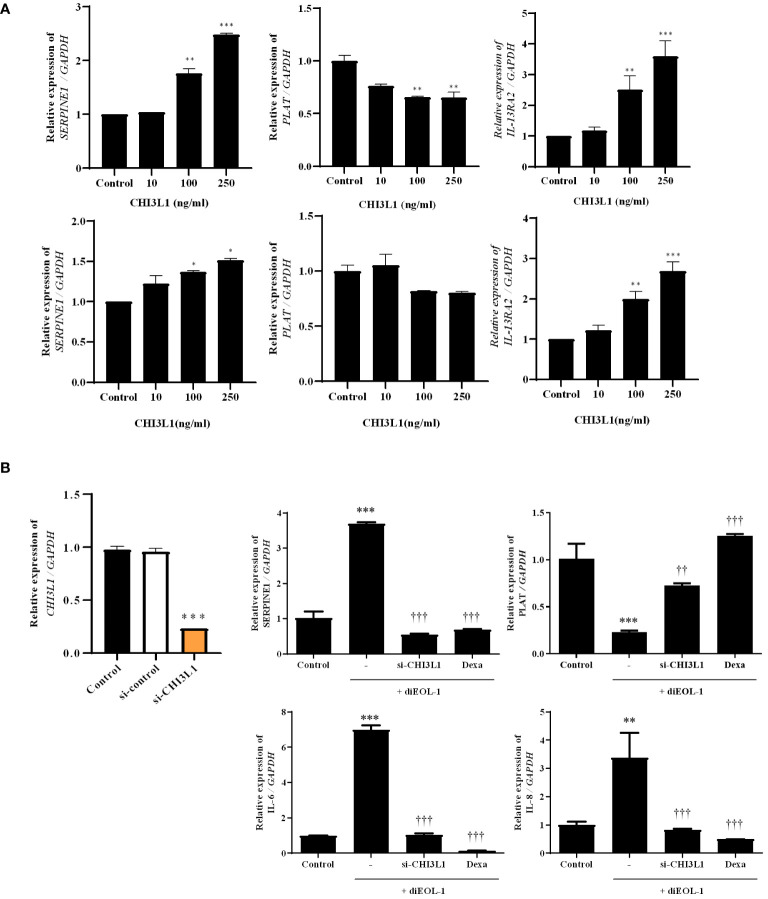
Effect of CHI3L1 on the fibrinolytic system of nasal structure cells **(A)** Expression results of SERPINE1, PLAT, IL-13RA2 following treatment with human recombinant CHI3L1 (0–250 ng/ml) using Real-time PCR. The upper panel shows results in nasal fibroblasts, while the lower panel is performed on ALI cells. *p < 0.05 vs. Control; **p < 0.01 vs. Control; ***p < 0.001 vs. Control. **(B)** Application in a 3D hybrid culture system after suppression of CHI3L1 secretion in diEOL-1 using si-CHI3L1. Gene expression of SERPINE1, PLAT, IL-6, and IL-8 confirmed using Real-time PCR. **p < 0.01 vs. Control; ***p < 0.001 vs. Control. ^††^p < 0.01 vs. diEOL-1; ^†††^p < 0.001 vs. diEOL-1;.

To investigate the effects of CHI3L1 on PAI-1, t-PA, and IL-13RA2 expression, nasal fibroblasts and PNECs were treated with huCHI3L1. Treatment with CHI3L1 significantly increased the expression of SERPINE1 and IL-13RA2 while inhibiting the expression of PLAT (**Figure 7A**). In a 3D hybrid culture system, we treated diEOL-1 cells with si-CHI3L1 to suppress CHI3L1 secretion, followed by co-culture. Consequently, the inhibition of CHI3L1 secretion improved the imbalance in the fibrinolytic system caused by diEOL-1. Furthermore, it showed a similar degree of improvement as with dexamethasone (**Figure 7B**).

## Discussion 

Chronic rhinosinusitis (CRS) is a multifaceted inflammatory disease that significantly impacts the global adult population. It is characterized by persistent inflammation of the nasal and paranasal sinuses, often leading to the formation of nasal polyps, particularly in eosinophilic ECRS (1). This study explores the interplay between eosinophils and the fibrinolytic system in CRS, emphasizing the role of eosinophil-derived CHI3L1 in modulating key components of this system and promoting fibrin deposition.

The fibrinolytic system is crucial for regulating blood clot formation and dissolution, suppressing coagulation within blood vessels, and dissolving clots when necessary. An imbalance in this system, characterized by elevated levels of PAI-1 and reduced levels of t-PA, can lead to fibrin deposition within tissues (10, 11). In ECRS, this imbalance contributes to tissue remodeling and the formation of nasal polyps (15, 16). Our study validated this imbalance within nasal polyp tissues, demonstrating that nasal fibroblasts and epithelial cells play a significant role in this dysregulation in response to eosinophils. These findings were confirmed through co-culture with differentiated and activated eosinophil cell lines and structural cells. Furthermore, we found that CHI3L1, secreted by differentiated eosinophil-like cells (diEOL-1), is instrumental in triggering this phenomenon. This discovery underscores the central role of CHI3L1 in contributing to fibrinolytic system imbalance and nasal polyp formation in ECRS.

By analyzing genomic data, we identified distinct gene expression patterns in CRSwNP tissues compared to those in normal mucosa tissues. Most normal tissues were included in the same cluster, which was characterized by high t-PA expression. In contrast, CRSwNP tissues were divided into several clusters based on gene expression patterns, with a group increasing PAI-1, u-PA, u-PAR, and CLC, accompanied by upregulation of Th2 cytokines, set as the target cluster for comparison with the normal cluster. DEG analysis between normal and target clusters revealed a significant upregulation of CCL8, CCL13, and CCL18 chemokines, which are associated with immune cell infiltration and immune response; IL-5, a Th2 cytokine; and CLC, a gene that is characteristically overexpressed in ECRS. In contrast, we observed repression in the expression of genes related to fluid production, such as STATH, CRSPI3, ROPN1, and LPO. Gene ontology results indicated that these changes reflected an overactive immune system, with increased leukocyte activation and migration in nasal tissues within the target cluster, along with diminished ion transport and homeostasis mechanisms. Additionally, we confirmed that immune response-related pathways, such as those involving platelet lineage cells, cytokines, chemokine signaling, Th1 and Th2 cell differentiation, and the formation of neutrophil extracellular traps, were activated. S. aureus infection, one of the causes of CRS, and complement and coagulation cascades, a pathway related to the fibrinolytic system that is the focus of this study, also showed a high correlation.

The data revealed a significant upregulation of PAI-1 and a concurrent downregulation of t-PA in nasal polyp tissues from ECRS patients. PAI-1, a serine protease inhibitor, impedes fibrinolysis by inhibiting t-PA leading to reduced plasmin generation and subsequent fibrin degradation. Elevated PAI-1 levels have been implicated in various fibrotic diseases, emphasizing its role in promoting tissue remodeling and fibrosis. In our study, PAI-1 expression was predominantly localized to fibroblasts and epithelial cells within the nasal mucosa, which are critical players in tissue repair and remodeling. Conversely, t-PA, which facilitates fibrinolysis by converting plasminogen to plasmin, was markedly reduced in nasal polyp tissues. This reduction in t-PA impairs fibrin degradation, contributing to fibrin accumulation and extracellular matrix (ECM) deposition. The imbalance between PAI-1 and t-PA creates a pro-fibrotic environment conducive to the development and persistence of nasal polyps in ECRS.

Allergic rhinitis, asthma, and aspirin sensitivity are eosinophilic diseases, and the treatment of the accompanying ECRS is difficult (31, 32). We analyzed whether this fibrinolytic imbalance was related to eosinophilic diseases. Aspirin is generally used as a platelet aggregation inhibitor that inhibits the activity and concentration of PAI-1. PAI-1 levels are significantly increased in aspirin-sensitive patients with CRS Therefore, controlling the overexpression of PAI-1 in patients with abnormal responses to aspirin may effectively improve disease progression. Patients with CRS and asthma showed suppressed tPA expression, but no corresponding elevation of PAI-1 was observed. Th2 cytokines can suppress the expression of t-PA in CRS, and since overexpression of Th2 cytokines is particularly observed in asthma, the excessive decrease in t-PA in CRS accompanied by asthma is likely to be an effect of Th2 cytokines (16).

Through scRNA analysis, it was confirmed that the major proteins that regulate the fibrinolytic system are secreted from mucosal structural cells. We performed *in vitro* experiments to determine whether eosinophils induce an imbalance in the fibrinolytic system. Although EOL-1 cells are eosinophilic, they are undifferentiated with myeloblastic characteristics. Therefore, we used these cells as a stand-in for primary eosinophils in our experiments after inducing differentiation with butyric acid and activating them with IL-3, IL-5, and GM-CSF. When we co-cultured diEOL-1 cells with primary nasal fibroblasts, we observed a significant increase in the expression of SERPINE1 and a significant decrease in PLAT expression. This effect was even more pronounced when diEOL-1 was activated and the culture included conditioned media. Similar results were observed in primary nasal epithelial cells (PNECs) cultured at an air-liquid interface (ALI). When PNECs were cocultured with diEOL-1, SERPINE1, CHI3L1, and IL-13RA2 expression increased, whereas PLAT expression decreased. These results suggest that not only the interaction with diEOL-1 but also the cytokines or chemokines secreted by diEOL-1 may be involved in the regulation of these fibrinolytic system-related genes. Furthermore, we observed simultaneous increases in the expression of CHI3L1 and its receptor IL-13RA2. Cytokine array analysis of diEOL-1 cells confirmed the overexpression of CHI3L1, which may influence the expression of CHI3L1 and IL-13RA2 in fibroblasts and PNECs. However, further studies are required to explore this hypothesis.

CHI3L1, a glycoprotein upregulated in eosinophils (differentiated into diEOL-1 cells), emerged as a critical regulator of the fibrinolytic system in ECRS. CHI3L1 significantly increased the expression of PAI-1 while decreasing t-PA levels in nasal fibroblasts and epithelial cells. The protein’s influence extends beyond simple modulation of fibrinolytic components; it appears to act as a mediator of Th2 inflammation, exacerbating the pro-fibrotic milieu within the nasal mucosa. The cytokine array analysis provided insights into the broader inflammatory landscape in which CHI3L1 operates. Th2 cytokines, including IL-4, IL-5, and IL-13, were significantly elevated in co-cultures of nasal fibroblasts and diEOL-1 cells. This Th2-skewed cytokine environment promotes eosinophil survival and activation, further amplifying the inflammatory and fibrotic responses. The correlation between Th2 cytokines and fibrinolytic system dysregulation highlights the intertwined nature of immune and fibrinolytic pathways in ECRS pathogenesis. We investigated the correlation between the fibrinolytic system and eosinophils by comparing PAI-1, t-PA, CLC, and fibrin expression in normal UP and nasal polyp tissues from NECRS and ECRS patients. While a decrease in t-PA and an increase in CLC were observed in nasal polyps, significant changes in PAI-1, u-PA, and u-PAR expression were evident only when comparing ECRSwNP and NECRSwNP tissues with normal UP. This suggests that fibrinolytic system imbalance is particularly characteristic of ECRS. To further explore the effects of CHI3L1, we developed a 3D hybrid culture system involving diEOL-1, fibroblast spheroids, and ALI cultures. Consistent with previous observations, co-culture with diEOL-1 led to increased SERPINE1 and CHI3L1 expression and suppressed PLAT expression. When human CHI3L1 was administered to nasal fibroblasts and PNECs, similar effects were observed, confirming the role of CHI3L1 in modulating the fibrinolytic system. Furthermore, si-CHI3L1 treatment of diEOL-1 cells in the 3D hybrid culture system effectively controlled the fibrinolytic imbalance, with effects comparable to dexamethasone.

Our findings suggest that targeting CHI3L1 could offer a novel therapeutic approach for managing ECRS. Inhibiting CHI3L1 restores the balance between PAI-1 and t-PA, reducing fibrin deposition and tissue remodeling. This approach may improve outcomes for patients who are refractory to conventional treatments. Further research is needed to explore the molecular mechanisms by which CHI3L1 modulates fibrinolytic components and to validate these findings in clinical settings. This study has several limitations. The use of *in vitro* models may not fully capture the complexity of *in vivo* conditions. Therefore, additional validation using primary eosinophils and animal models is required. Moreover, the heterogeneity of CRS necessitates personalized approaches to treatment. Understanding how CHI3L1 modulation affects different subsets of CRS patients will be crucial for developing targeted therapies.

## Conclusions

Our findings shed new light on the complex interplay among eosinophils, nasal fibroblasts, and epithelial cells in the imbalance of the fibrinolytic system within nasal polyps during CRS. In conclusion, our study identifies CHI3L1 as a key mediator of fibrinolytic system dysregulation in ECRS, promoting fibrin deposition through the upregulation of PAI-1 and downregulation of t-PA. These insights contribute to our understanding of the intricate pathophysiological mechanisms in chronic rhinosinusitis and can inform the development of future therapeutic strategies.

## Data availability statement

The datasets presented in this study can be found in online repositories. The names of the repository/repositories and accession number(s) can be found below: https://www.ncbi.nlm.nih.gov/geo/, GSE23552, https://www.ncbi.nlm.nih.gov/geo/, GSE36830, https://www.ncbi.nlm.nih.gov/geo/, GSE179265, https://ngdc.cncb.ac.cn/gsa-human/browse/HRA000772, HRA000772.

## Ethics statement

The studies involving humans were approved by Institutional Review Board of Korea University Medical Center. The participants provided their written informed consent to participate in this study. The animal study was approved by Institutional Review Board of Korea University Medical Center. The study was conducted in accordance with the local legislation and institutional requirements.

## Author contributions

H-WY: Writing – review & editing, Writing – original draft, Visualization, Validation, Supervision, Project administration, Investigation, Formal Analysis, Data curation, Conceptualization. J-HP: Writing – review & editing, Conceptualization. J-MS: Writing – review & editing, Conceptualization. H-GS: Writing – review & editing, Investigation, Data curation, Conceptualization. T-HK: Writing – review & editing, Conceptualization. S-HL: Writing – review & editing, Conceptualization. I-HP: Writing – review & editing, Writing – original draft, Validation, Supervision, Project administration, Data curation, Conceptualization.
