# Supplementary figures and images for: CHI3L1 on fibrinolytic system imbalance in chronic rhinosinusitis with nasal polyp
Source: Front Immunol. 2024 Jun 21;15:1410948. doi: 10.3389/fimmu.2024.1410948 (PMC11224434; doi:10.3389/fimmu.2024.1410948)

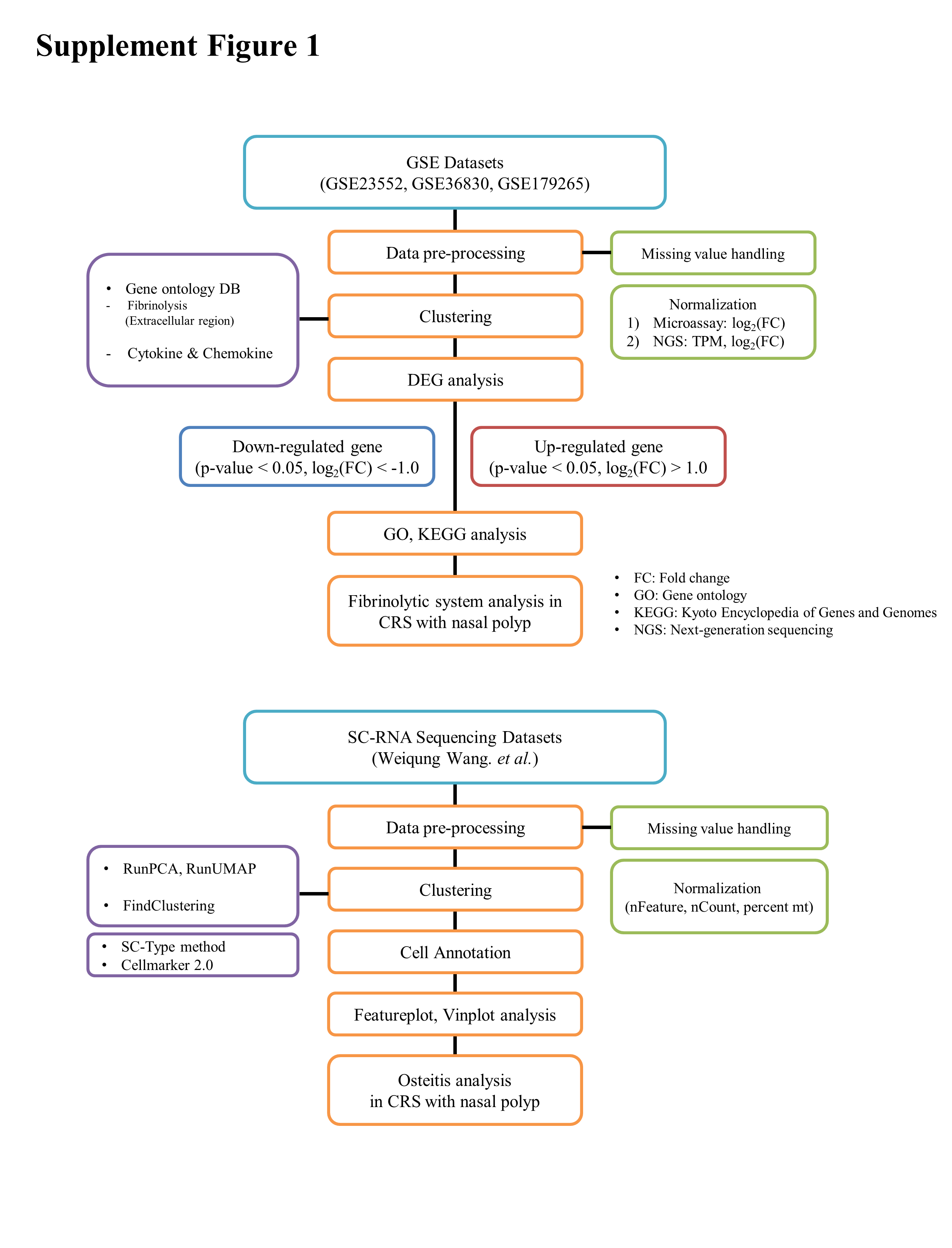

Supplement: Supplementary file 1 [file Image_1.tif]

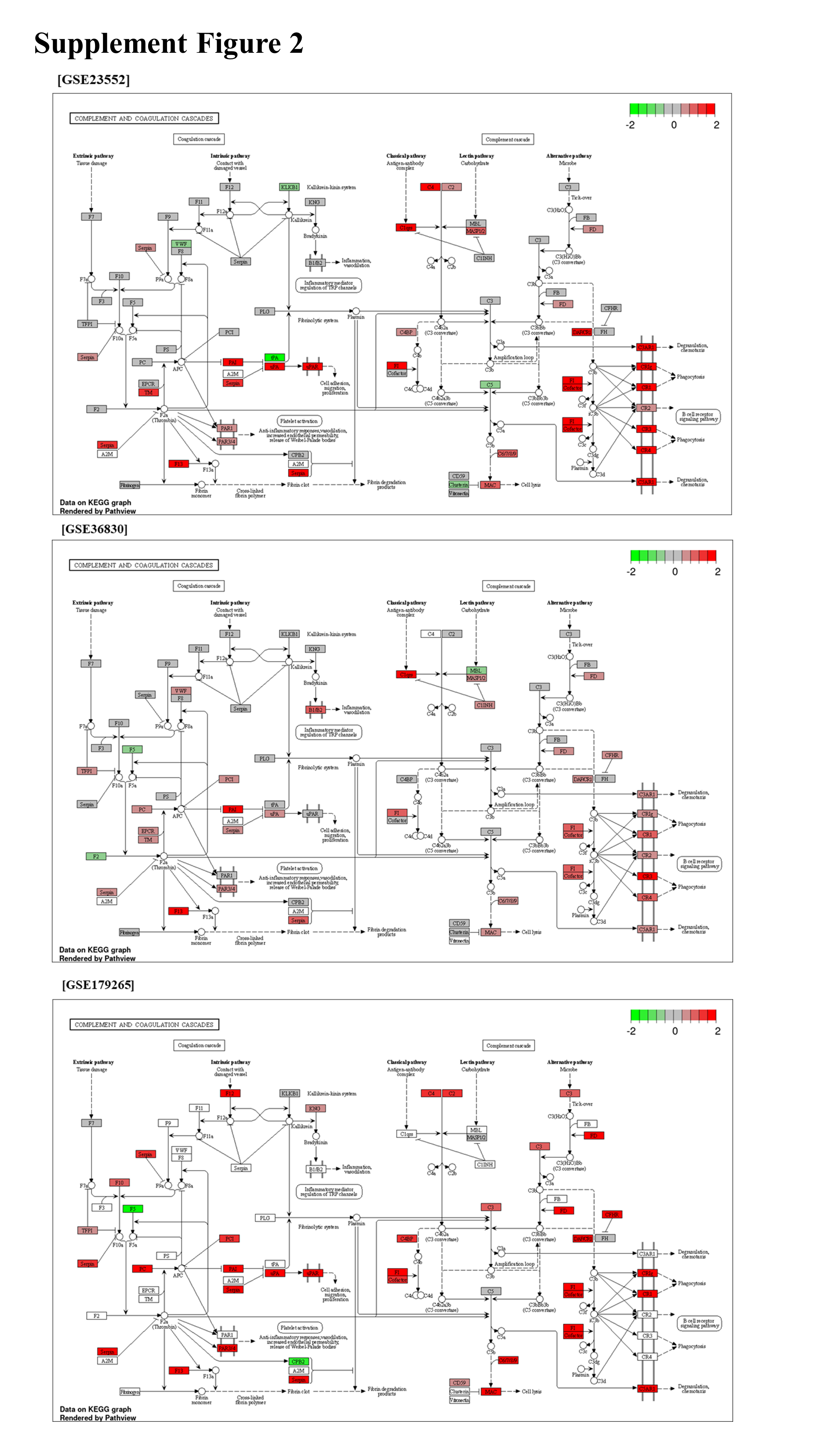

Supplement: Supplementary file 2 [file Image_2.tif]

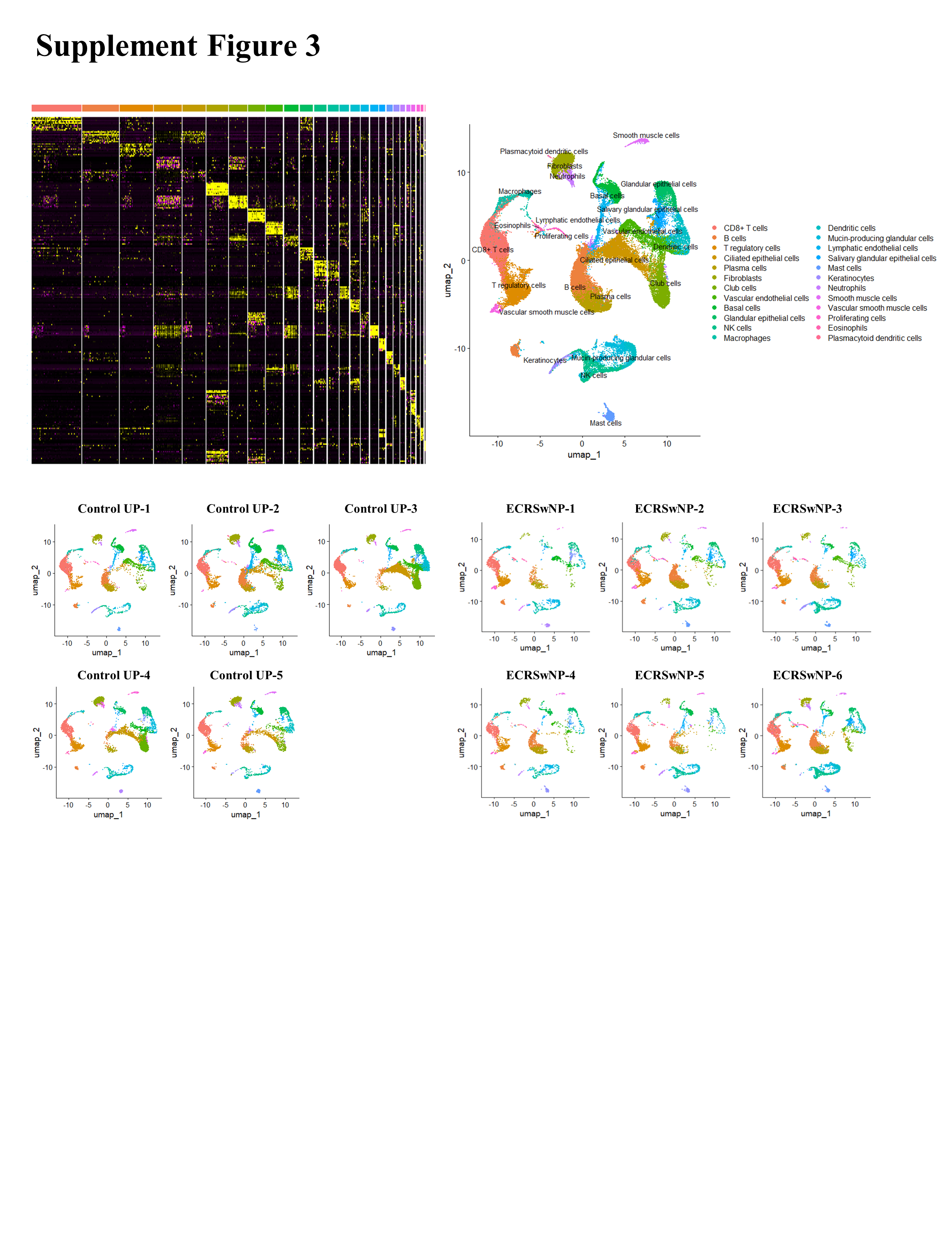

Supplement: Supplementary file 3 [file Image_3.tif]
